# Angioinvasion severity and admission GCS predict outcomes in invasive central nervous system fungal infections

**DOI:** 10.3389/fcimb.2026.1915699

**Published:** 2026-08-13

**Authors:** Yushu Jiang, Shuhua Dai, Rui Pang, Lingzhi Qin, Milan Zhang, Huiqin Liu, Xiaojuan Wang, Jiewen Zhang, Wei Li

**Affiliations:** 1 Department of Neurology, Henan Joint International Research Laboratory of Accurate Diagnosis, Treatment, Research And Development, Henan Provincial People’s Hospital, People’s Hospital of Zhengzhou University, Zhengzhou, Henan, China; 2 Department of Neurology, Zhoukou Central Hospital, Zhoukou, Henan, China

**Keywords:** angioinvasion, aspergillosis, central nervous system fungal infections, Glasgow Coma Scale, modified Rankin Scale, mucormycosis

## Abstract

**Background:**

Central nervous system (CNS) invasive fungal infections caused by *Aspergillus* spp. and Mucorales are associated with high mortality and severe neurological disability, but predictors of functional outcome remain poorly defined. We compared clinical features, neuroimaging patterns, and outcomes among rhino-orbital-cerebral mucormycosis (ROCM), rhino-cerebral aspergillosis (RA), and cerebral aspergillosis (CA), and identified prognostic factors for mortality and functional outcome.

**Methods:**

We performed a retrospective cohort study of 57 adults with proven or probable invasive CNS fungal infection (28 ROCM, 13 RA, 16 CA) treated between January 2018 and April 2025. An exploratory imaging-based angioinvasion severity score (0–3) was constructed from three radiographic markers: internal carotid artery involvement, cerebral infarction, and intracranial hemorrhage. The primary outcome was all-cause mortality during follow-up; 90-day mortality was also assessed. The secondary outcome was 90-day unfavorable functional outcome, defined as modified Rankin Scale (mRS) 3–6. Multivariable analyses used Cox regression, Firth penalized logistic regression, and proportional odds ordinal logistic regression.

**Results:**

ROCM had the highest 90-day mortality (43% vs. 7.7% in RA and 6.3% in CA; *p* = 0.009) and the lowest rate of favorable 90-day functional outcome (50% vs. 92% and 81%; *p* = 0.012). Moderate-to-severe angioinvasion (score ≥2) was associated with worse 90-day ordinal disability (common OR 8.61, 95% CI 1.87–39.58; *p* = 0.006). Lower admission Glasgow Coma Scale (GCS) score independently predicted mortality (adjusted HR 0.77, 95% CI 0.67–0.88; *p* < 0.001) and unfavorable functional outcome (adjusted OR 0.68, 95% CI 0.36–0.91; *p* = 0.006). After adjustment for angioinvasion burden and GCS, fungal phenotype was not independently associated with prognosis. The prediction model for unfavorable functional outcome showed good discrimination (AUC 0.888).

**Conclusions:**

Higher angioinvasion severity was associated with worse neurological functional outcome, whereas admission GCS independently predicted both mortality and unfavorable functional outcome. These findings may assist early risk stratification and treatment planning in invasive CNS fungal infection.

## 1 Introduction

Central nervous system (CNS) invasive fungal infections caused by Aspergillus species and the order Mucorales are rare but devastating diseases (Chantelot et al., 2026; Kontoyiannis and Walsh, 2026). Although these infections account for only a small fraction of all CNS infections, they are disproportionately lethal (Bongomin et al., 2017). Mortality rates for CNS aspergillosis range from 50% to 85–99% in classic immunocompromised hosts (Meena et al., 2021), and up to 80% for CNS mucormycosis even with optimal therapy (Pai et al., 2021). The incidence has been rising globally, driven by an expanding population of immunocompromised individuals—including those with hematological malignancies, hematopoietic stem cell or solid organ transplant recipients, patients receiving newer immunosuppressive therapies (e.g., BTK inhibitors, CAR-T cells), and importantly, the growing burden of poorly controlled diabetes mellitus (Abdolalizadeh et al., 2020; Gouzien et al., 2024). This epidemiological shift, further amplified by the COVID-19 pandemic, has made CNS invasive fungal infections an increasingly pressing clinical concern (Singh and Vishnu, 2021). Given the high morbidity and mortality, early prognostic stratification remains a critical but challenging clinical need.

Prior studies on invasive CNS fungal infections have largely focused on single pathogens, with mucormycosis and aspergillosis analyzed separately in distinct cohorts. According to the correlation between intracranial lesions and paranasal sinus lesions, previous reports have often classified CNS aspergillosis into two types: rhino-cerebral aspergillosis (RA) and cerebral aspergillosis (CA) (Siddiqui et al., 2004). However, RA and rhino-orbital-cerebral mucormycosis (ROCM) share similar clinical and radiological presentations, making direct comparisons both feasible and necessary (Tripathi and Mohindra, 2018; Jeong et al., 2019). While a pathogen-centric approach has advanced our understanding of each disease individually, it has inadvertently created critical knowledge gaps. Comparative data across different fungal etiologies—particularly regarding neuroimaging patterns, the extent of fungal angioinvasion and its associated cerebrovascular complications (hereafter referred to as angioinvasion burden), and functional outcomes—remain sparse. Angioinvasion burden reflects the cumulative degree of fungal-induced neurovascular injury, including large-vessel invasion, cerebral infarction, and intracranial hemorrhage, which are considered key manifestations of invasive fungal cerebrovascular disease (Kontoyiannis and Walsh, 2026; La Porte et al., 2026). Moreover, predictors of neurological functional outcome (e.g., modified Rankin Scale [mRS] score) have been poorly characterized, with most reports focusing on mortality alone, and ordinal analysis of disability across the full mRS spectrum has not been systematically evaluated. Whether the worse prognosis traditionally attributed to ROCM arises from the fungal species itself or from its anatomically determined greater burden of large-vessel angioinvasion remains an unresolved question (Serris et al., 2022; La Porte et al., 2026).

To address these gaps, we performed a retrospective cohort study of 57 adult patients with proven or probable invasive CNS fungal infection (ROCM, RA, and CA) to compare clinical characteristics, neuroimaging patterns, mortality, and functional outcomes. We introduced a novel angioinvasion severity score (0–3) based on radiographic markers of fungal cerebrovascular involvement (internal carotid artery involvement [ICA], cerebral infarction, and intracranial hemorrhage), and systematically evaluated mortality and short- and intermediate-term functional outcome using both binary and ordinal mRS analyses. Our objectives were to identify independent predictors of survival and neurological disability, characterize phenotype-specific angioinvasion patterns, and assess the discriminative performance of the angioinvasion severity score for prognostic stratification.

## 2 Materials and methods

### 2.1 Study design and patient population

This retrospective cohort study was conducted at Henan Provincial People’s Hospital and included consecutive adult patients with suspected mucormycosis or aspergillosis involving the CNS who were admitted between January 2018 and April 2025. This study was performed in accordance with the principles of the Declaration of Helsinki. Approval was granted by the Henan Provincial Peoples Hospital Ethics Committee, China (approval No. 2021-04). The requirement for informed consent for the retrospective analysis of anonymized clinical data was waived by the ethics committee.

To ensure cohort homogeneity and minimize diagnostic heterogeneity, patients were rigorously screened according to predefined inclusion and exclusion criteria. Eligible patients were required to meet all of the following inclusion criteria: (1) age ≥18 years; (2) clinical manifestations together with neuroimaging and/or sinonasal imaging findings suggestive of sino-cranial or CNS-invasive mucormycosis or aspergillosis; (3) fulfillment of the criteria for proven or probable invasive mucormycosis or aspergillosis according to the revised consensus definitions of the European Organization for Research and Treatment of Cancer and the Mycoses Study Group Education and Research Consortium (EORTC/MSGERC) (Donnelly et al., 2020). Patients with noninvasive fungal disease, CNS infections caused by other pathogens, alternative neurological diagnoses explaining imaging abnormalities, or incomplete clinical/follow-up data were excluded.

**Figure 1:**
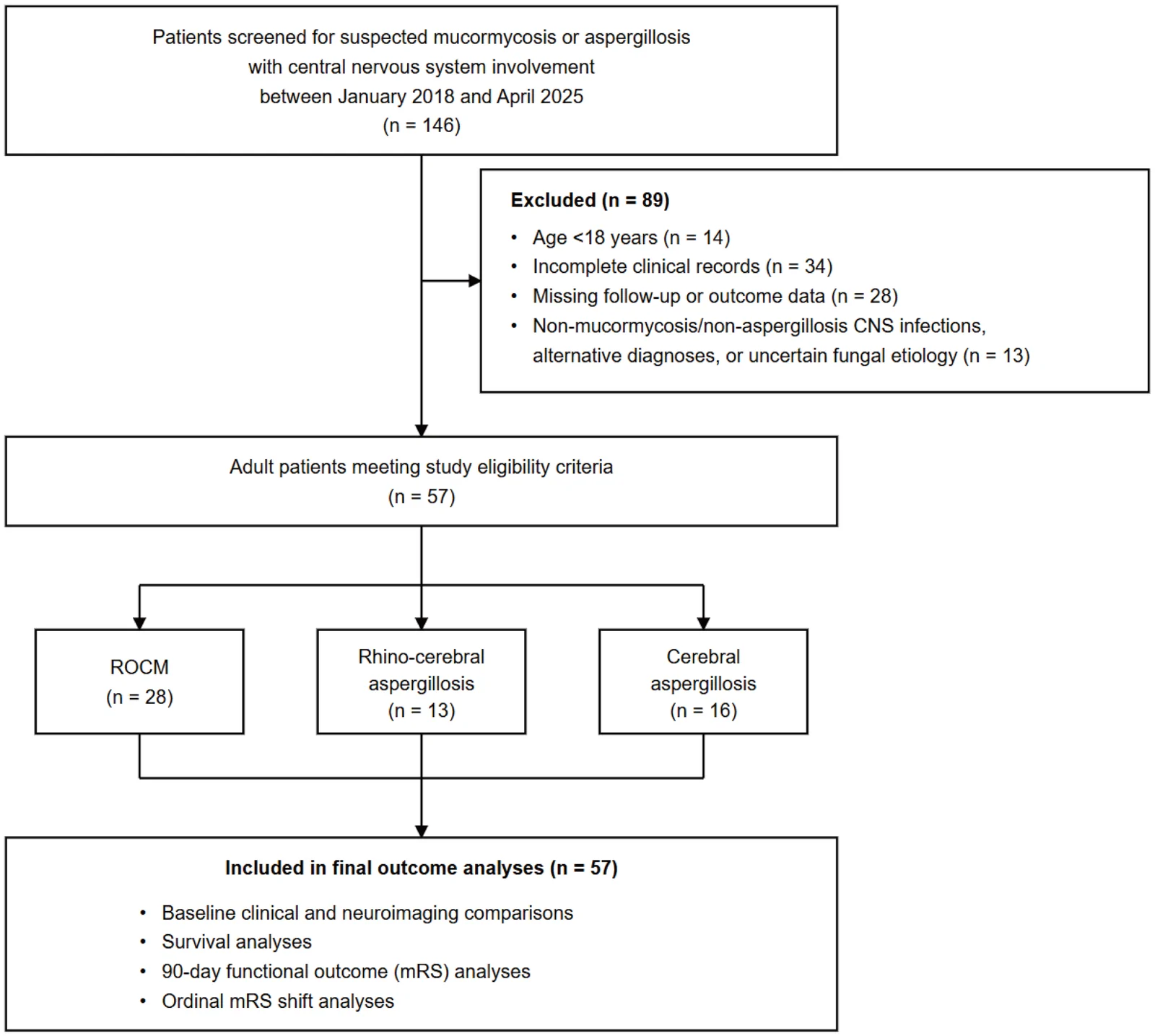
Study flowchart of patient selection and analysis population. Adult patients with mucormycosis or aspergillosis involving the central nervous system who were screened between January 2018 and April 2025 are shown. After exclusion of patients with incomplete clinical records, missing follow-up or outcome data, age <18 years, or uncertain fungal diagnoses, 57 patients were included in the final cohort. Patients were categorized into three groups: ROCM, RA, and CA. All included patients underwent survival and functional outcome analyses, including ordinal mRS shift analysis at 90 days.

A total of 146 patients were screened, of whom 57 met the study criteria and were included in the final cohort. Patients were categorized into three infection phenotypes according to anatomical involvement and fungal etiology: ROCM, RA, and CA. The study flowchart is presented in **Figure 1**.

### 2.2 Data collection

Clinical data were extracted from electronic medical records using a standardized data collection form. Collected variables included: demographic characteristics, underlying comorbidities, clinical manifestations, laboratory findings, cerebrospinal fluid parameters, neuroimaging characteristics, antifungal treatment strategies, surgical interventions and clinical outcomes. Neurological severity at admission was assessed using the Glasgow Coma Scale (GCS). Functional outcome was evaluated using the mRS at 90 days and 180 days after diagnosis.

### 2.3 Neuroimaging assessment and angioinvasion severity classification

All available neuroimaging studies, including computed tomography (CT) and magnetic resonance imaging (MRI), were reviewed for evidence of fungal angioinvasion and CNS injury. MRI protocols typically included T1-weighted, T2-weighted, fluid-attenuated inversion recovery (FLAIR), diffusion-weighted imaging (DWI), and contrast-enhanced sequences. CT imaging included non-contrast and, when available, contrast-enhanced scans. Magnetic resonance angiography (MRA) was performed in patients with suspected vascular involvement as part of routine clinical evaluation to assess intracranial arterial integrity, particularly the ICA.

Imaging variables of interest included vascular invasion, ICA involvement, cavernous sinus involvement, cerebral infarction, intracranial hemorrhage, and major vessel stenosis or occlusion. ICA involvement was defined as radiological evidence of arterial stenosis or occlusion on MRA or contrast-enhanced MRI. Cerebral infarction was defined as restricted diffusion on DWI consistent with acute ischemia. Intracranial hemorrhage was identified on CT or susceptibility-sensitive MRI sequences. All imaging studies were independently reviewed by two neuroradiologists blinded to clinical outcomes, and discrepancies were resolved by consensus.

Angioinvasion burden was defined as the cumulative extent of fungal-induced cerebrovascular involvement, including major-vessel invasion and its downstream ischemic and hemorrhagic complications. To quantify angioinvasion burden, we developed an imaging-based angioinvasion severity score as a pragmatic composite measure. The three score components—internal carotid artery involvement, cerebral infarction, and intracranial hemorrhage—were selected *a priori* based on their biological plausibility and previously reported relevance in angioinvasive fungal infections. Each component was assigned 1 point if present, yielding a total score ranging from 0 to 3.

Angioinvasion severity was subsequently categorized as:

- none (score = 0),
- mild (score = 1),
- moderate-to-severe (score ≥2).

This classification framework was designed to reflect the severity of angioinvasion burden rather than fungal phenotype alone.

### 2.4 Outcome definitions

The primary outcome was all-cause mortality during the follow-up period, with follow-up censored on August 31, 2025. The secondary outcome was unfavorable functional outcome at 90 days, defined as a mRS score of 3–6. As an exploratory analysis, ordinal shift analysis was performed using the full 90-day mRS distribution. Additional medium-term functional outcome analyses were performed at 180 days, including both dichotomized unfavorable outcome (mRS 3–6) and ordinal shift analysis across the full 180-day mRS distribution.

### 2.5 Statistical analysis

Categorical variables are presented as counts and percentages and were compared using Fisher’s exact test. Continuous variables are presented as median with interquartile range or mean with standard deviation, as appropriate, and were compared using the Kruskal-Wallis test or Mann-Whitney U test.

Time-to-death analyses were performed using Kaplan-Meier survival analysis and Cox proportional hazards regression. Survival time was calculated from diagnosis until death or administrative censoring on August 31, 2025. All 139 baseline variables (**Supplementary Table 1**) were screened by univariable Cox regression; treatment-related and post-baseline variables were excluded *a priori*. Variables with p < 0.05 in univariable analysis, together with clinically relevant covariates (age, sex, infection phenotype, admission GCS), were considered for inclusion in multivariable Cox models. Firth penalized Cox regression was applied when separation occurred.

For analyses of 90-day unfavorable functional outcome (mRS 3–6), univariable logistic regression was initially performed. Given the limited sample size and potential sparse-data bias, multivariable analyses were conducted using Firth penalized logistic regression. Model discrimination was evaluated using receiver operating characteristic (ROC) curve analysis. Additional analyses of 180-day unfavorable functional outcome were performed using the same modeling strategy.

Ordinal functional outcome analysis was performed using proportional odds ordinal logistic regression with the full 90-day mRS distribution as the dependent variable. Additional ordinal shift analysis was also conducted using the full 180-day mRS distribution. The proportional odds assumption was assessed using nominal tests.

All analyses were conducted using complete-case data for variables included in each model. Effect estimates are reported as hazard ratios (HRs) or odds ratios (ORs) with 95% confidence intervals (CIs). All statistical tests were two-sided, and *p* < 0.05 was considered statistically significant. Statistical analyses were performed using R software (version 4.5.2; R Foundation for Statistical Computing, Vienna, Austria).

## 3 Results

### 3.1 Cohort selection and baseline clinical characteristics

A total of 146 patients with suspected mucormycosis or aspergillosis involving the CNS were screened between January 2018 and April 2025. After exclusion of patients aged <18 years, those with incomplete records or unavailable follow-up, and cases with uncertain fungal etiologies or alternative diagnoses, 57 patients were included in the final cohort (**Figure 1**). Among them, 28 patients were classified as ROCM, 13 as RA, and 16 as CA.

Baseline clinical characteristics are summarized in **Table 1**. Diabetes mellitus was the predominant underlying condition and was particularly enriched in ROCM (26/28, 93% vs. 9/13, 69% vs. 6/16, 38%, *p* < 0.001). Diabetic ketoacidosis occurred exclusively in ROCM (10/28, 36%, *p* = 0.002), whereas classical immunocompromising conditions, including hematologic malignancy, transplantation, corticosteroid exposure, and immunosuppressant use, were more frequently observed in aspergillosis.

**Table 1:** Baseline clinical characteristics across infection phenotypes.

Variable	N	Overall N = 57^1^	ROCM N = 28^1^	Rhino-cerebral Aspergillosis N = 13^1^	Cerebral Aspergillosis N = 16^1^	p-value^2^
Demographics and baseline characteristics						
Age (years)	57	61.0 (49.0-69.0)	62.0 (53.5-69.0)	64.0 (56.0-73.0)	54.5 (38.0-68.5)	0.317
Sex	57					0.634
*Female*		21 (37%)	9 (32%)	6 (46%)	6 (38%)	
*Male*		36 (63%)	19 (68%)	7 (54%)	10 (63%)	
Onset-to-admission interval (days)	57	16.0 (8.0-46.0)	14.5 (8.0-26.5)	62.0 (11.0-189.0)	21.0 (9.5-44.5)	0.240
Host factors						
Diabetes mellitus	57	41 (72%)	26 (93%)	9 (69%)	6 (38%)	**<0.001**
Diabetic ketoacidosis	57	10 (18%)	10 (36%)	0 (0%)	0 (0%)	**0.002**
Hematologic malignancy	57	3 (5.3%)	1 (3.6%)	0 (0%)	2 (13%)	0.426
Transplantation history	57	2 (3.5%)	0 (0%)	1 (7.7%)	1 (6.3%)	0.254
Corticosteroid use	57	1 (1.8%)	0 (0%)	0 (0%)	1 (6.3%)	0.509
Immunosuppressant use	57	3 (5.3%)	0 (0%)	1 (7.7%)	2 (13%)	0.125
Liver cirrhosis	57	2 (3.5%)	0 (0%)	1 (7.7%)	1 (6.3%)	0.254
CCI category	57					0.247
*None*		7 (12%)	1 (3.6%)	3 (23%)	3 (19%)	
*Mild*		33 (58%)	17 (61%)	8 (62%)	8 (50%)	
*Severe*		17 (30%)	10 (36%)	2 (15%)	5 (31%)	
Key clinical manifestations						
Fever	57	40 (70%)	22 (79%)	7 (54%)	11 (69%)	0.248
Headache	57	53 (93%)	27 (96%)	13 (100%)	13 (81%)	0.120
Palatal necrosis	57	8 (14%)	8 (29%)	0 (0%)	0 (0%)	**0.009**
Facial swelling	57	30 (53%)	26 (93%)	4 (31%)	0 (0%)	**<0.001**
Blindness	57	31 (54%)	20 (71%)	9 (69%)	2 (13%)	**<0.001**
Ophthalmoplegia	57	34 (60%)	24 (86%)	7 (54%)	3 (19%)	**<0.001**
Hemiplegia	57	15 (26%)	11 (39%)	1 (7.7%)	3 (19%)	0.088
Consciousness disturbance	57	19 (33%)	11 (39%)	1 (7.7%)	7 (44%)	0.080
Admission GCS	57	15.0 (12.0-15.0)	15.0 (9.0-15.0)	15.0 (15.0-15.0)	15.0 (12.5-15.0)	0.106
Laboratory findings at admission						
Admission glucose (mmol/L)	57	7.6 (5.9-12.6)	12.1 (7.5-15.9)	6.4 (5.6-8.9)	6.1 (5.2-6.9)	**<0.001**
HbA1c (%)	53	8.6 (6.2-10.6)	10.4 (9.3-12.4)	7.4 (6.1-9.9)	6.2 (5.4-7.6)	**<0.001**
White blood cell count (×10^9^/L)	57	8.5 (6.6-14.1)	11.6 (7.2-17.6)	8.5 (6.6-11.7)	6.9 (5.2-9.9)	0.060
Neutrophil count (×10^9^/L)	57	6.7 (4.4-12.3)	9.7 (5.4-16.0)	6.2 (4.8-8.6)	4.7 (3.6-8.2)	0.051
Lymphocyte count (×10^9^/L)	57	1.2 (0.9-1.7)	1.0 (0.8-1.6)	1.2 (1.0-1.9)	1.2 (0.9-1.6)	0.408
CRP (mg/L)	55	32.8 (8.8-103.7)	98.3 (32.8-137.1)	18.5 (7.6-46.1)	6.3 (2.3-22.3)	**<0.001**
CSF white blood cell count (×10^6^/L)	39	38.0 (5.0-160.0)	26.5 (5.0-84.0)	22.0 (4.0-181.0)	71.0 (10.0-242.0)	0.552
CSF protein (g/L)	40	0.7 (0.4-1.0)	0.6 (0.4-1.2)	0.8 (0.4-0.9)	0.7 (0.5-1.3)	0.752
Microbiological and pathological findings						
Positive tissue culture	39	4 (10%)	2 (8.0%)	1 (9.1%)	1 (33%)	0.338
Positive histopathology	38	30 (79%)	22 (92%)	6 (55%)	2 (67%)	**0.024**
Positive NGS result	34	29 (85%)	12 (80%)	5 (71%)	12 (100%)	0.186
Serum GM index	52	0.2 (0.1-0.5)	0.1 (0.1-0.2)	0.3 (0.2-0.6)	0.5 (0.2-1.0)	**<0.001**
Diagnostic classification						
Proven diagnosis	57	18 (32%)	9 (32%)	7 (54%)	2 (13%)	0.064
Probable diagnosis	57	39 (68%)	19 (68%)	6 (46%)	14 (88%)	0.064

^1^n (%); Median (Q1-Q3).

^2^Fisher’s exact test; Kruskal-Wallis rank sum test.

CCI, Charlson Comorbidity Index; GCS, Glasgow Coma Scale; CRP, C-reactive protein; CSF, cerebrospinal fluid; NGS, next-generation sequencing; GM, galactomannan.

Bold values indicate statistically significant differences (p < 0.05).

Clinical presentation reflected distinct anatomical patterns of disease involvement. ROCM was characterized by prominent sino-orbital manifestations, including facial swelling (26/28, 93% vs. 4/13, 31% vs. 0/16, 0%; *p* < 0.001), ophthalmoplegia (24/28, 86% vs. 7/13, 54% vs. 3/16, 19%; p<0.001), blindness (20/28, 71% vs. 9/13, 69% vs. 2/16, 13%; *p* < 0.001), and palatal necrosis (8/28, 29% vs. 0 vs. 0; *p* = 0.009). Neurological manifestations, including hemiplegia (11/28, 39% vs. 1/13, 7.7% vs. 3/16, 19%; *p* = 0.088) and consciousness disturbance (11/28, 39% vs. 1/13, 7.7% vs. 7/16, 44%; *p* = 0.080), showed no statistically significant differences among infection phenotypes.

Laboratory findings also differed substantially across infection phenotypes. ROCM was associated with markedly higher glucose, HbA1c, and inflammatory marker levels (all *p* < 0.001), consistent with severe metabolic dysregulation. In contrast, serum galactomannan indices were highest in CA (*p* < 0.001). Histopathological confirmation was more frequently achieved in ROCM (22/24, 92%) compared with RA (6/11, 55%) and CA (2/3, 67%) (*p* = 0.024). In contrast, positive tissue culture (2/25, 8.0% vs. 1/11, 9.1% vs. 1/3, 33%; *p* = 0.338) and NGS positivity (12/15, 80% vs. 5/7, 71% vs. 12/12, 100%; *p* = 0.186) were comparable among groups.

Species-level fungal identification was available in 29 patients. Among these, the most frequently identified species were *Aspergillus flavus* (n=9) and *Rhizopus oryzae* (n=7), followed by *Aspergillus fumigatus* (n=4), *Rhizopus delemar* (n=3), *Aspergillus terreus* (n=3), *Rhizopus arrhizus* (n=1), *Rhizopus microsporus* (n=1), and *Aspergillus oryzae* (n=1).

Regarding the diagnostic classification, 18 patients (32%) met the criteria for proven CNS fungal infection and 39 (68%) were classified as probable. Of the 30 patients with positive histopathology, the majority (27/30, 90.0%) were obtained from sinonasal tissue rather than brain parenchyma (3/30, 10.0%). According to the EORTC/MSGERC criteria, histopathological evidence of fungal elements from sinonasal tissue supports a diagnosis of probable invasive fungal disease but does not fulfill the criteria for proven CNS infection. These differences in specimen origin explain the higher rate of positive histopathology (30/38, 79%) relative to the proven diagnosis rate (18/57, 32%). A detailed breakdown of diagnostic test results by specimen type and infection phenotype is provided in **Supplementary Table 2**.

### 3.2 Neuroimaging characteristics across infection phenotypes

Neuroimaging patterns differed substantially across infection phenotypes and suggested distinct routes of intracranial involvement (**Table 2**). ROCM predominantly demonstrated a contiguous sino-orbital-skull base extension pattern, whereas CA more frequently exhibited imaging features suggestive of hematogenous dissemination. Compared with CA, ROCM showed significantly higher frequencies of sinonasal involvement (28/28, 100% vs. 7/16, 44%; *p* < 0.001), orbital involvement (24/28, 86% vs. 1/16, 6.3%; *p* < 0.001), skull-base involvement (16/28, 57% vs. 0/16, 0%; *p* < 0.001), and cavernous sinus involvement (17/28, 61% vs. 2/16, 13%; *p* = 0.005).

**Table 2:** Neuroimaging characteristics across infection phenotypes.

Variable	N	Overall N = 57^1^	ROCM N = 28^1^	Rhino-cerebral Aspergillosis N = 13^1^	Cerebral Aspergillosis N = 16^1^	p-value^2^
Sinonasal/orbital extension						
Pansinusitis	57	32 (56%)	23 (82%)	8 (62%)	1 (6.3%)	**<0.001**
Sinonasal involvement	57	47 (82%)	28 (100%)	12 (92%)	7 (44%)	**<0.001**
Orbital involvement	57	31 (54%)	24 (86%)	6 (46%)	1 (6.3%)	**<0.001**
Cavernous sinus	57	23 (40%)	17 (61%)	4 (31%)	2 (13%)	**0.005**
Skull base	57	18 (32%)	16 (57%)	2 (15%)	0 (0%)	**<0.001**
Pterygopalatine fossa	57	30 (53%)	27 (96%)	3 (23%)	0 (0%)	**<0.001**
Infratemporal fossa	57	27 (47%)	25 (89%)	2 (15%)	0 (0%)	**<0.001**
Neurovascular invasion						
ICA involvement	57	18 (32%)	14 (50%)	3 (23%)	1 (6.3%)	**0.007**
Cerebral infarction	57	23 (40%)	16 (57%)	5 (38%)	2 (13%)	**0.013**
Intracranial hemorrhage	57	7 (12%)	4 (14%)	2 (15%)	1 (6.3%)	0.763
Angioinvasion score category	57					**0.014**
*None*		30 (53%)	9 (32%)	8 (62%)	13 (81%)	
*Mild*		9 (16%)	5 (18%)	2 (15%)	2 (13%)	
*Moderate-severe*		18 (32%)	14 (50%)	3 (23%)	1 (6.3%)	
Intracranial dissemination						
Brain abscess	57	16 (28%)	7 (25%)	1 (7.7%)	8 (50%)	**0.040**
Number of lesions	57					0.125
*None*		41 (72%)	21 (75%)	12 (92%)	8 (50%)	
*Single*		7 (12%)	3 (11%)	1 (7.7%)	3 (19%)	
*Multiple*		9 (16%)	4 (14%)	0 (0%)	5 (31%)	
Bilateral lesions	16					0.119
*Unilateral*		10 (63%)	6 (86%)	1 (100%)	3 (38%)	
*Bilateral*		6 (38%)	1 (14%)	0 (0%)	5 (63%)	
Meningeal involvement	57	32 (56%)	14 (50%)	9 (69%)	9 (56%)	0.573
Hematogenous dissemination	57	7 (12%)	1 (3.6%)	0 (0%)	6 (38%)	**0.002**
Encephalitis-like lesion	57	23 (40%)	12 (43%)	4 (31%)	7 (44%)	0.777

^1^n (%); Median (Q1-Q3).

^2^Fisher’s exact test; Kruskal-Wallis rank sum test.

ICA, internal carotid artery.

Bold values indicate statistically significant differences (p < 0.05).

In contrast, CA demonstrated imaging characteristics consistent with more disseminated intracranial involvement. Brain abscess formation was more frequent in CA than ROCM (8/16, 50% vs. 7/28, 25%; *p* = 0.040). Multifocal intracranial lesions were observed in 5/16 (31%) CA patients compared with 4/28 (14%) ROCM and 0/13 (0%) RA patients, although the difference did not reach statistical significance (*p* = 0.125). These findings are compatible with the previously described tendency of Aspergillus infection to cause hematogenous dissemination and intracranial abscess formation; however, the present imaging-based analysis cannot directly determine the biological route of fungal spread.

Markers of angioinvasive disease, defined *a priori* as ICA involvement, cerebral infarction, and intracranial hemorrhage, were significantly enriched in ROCM. ICA involvement occurred in 14/28 (50%) ROCM, 3/13 (23%) RA, and 1/16 (6.3%) CA patients (*p* = 0.007). Cerebral infarction occurred in 16/28 (57%), 5/13 (38%), and 2/16 (13%), respectively (*p* = 0.013), whereas intracranial hemorrhage did not differ significantly among groups (4/28, 14% vs. 2/13, 15% vs. 1/16, 6.3%; *p* = 0.763).

**Figure 2:**
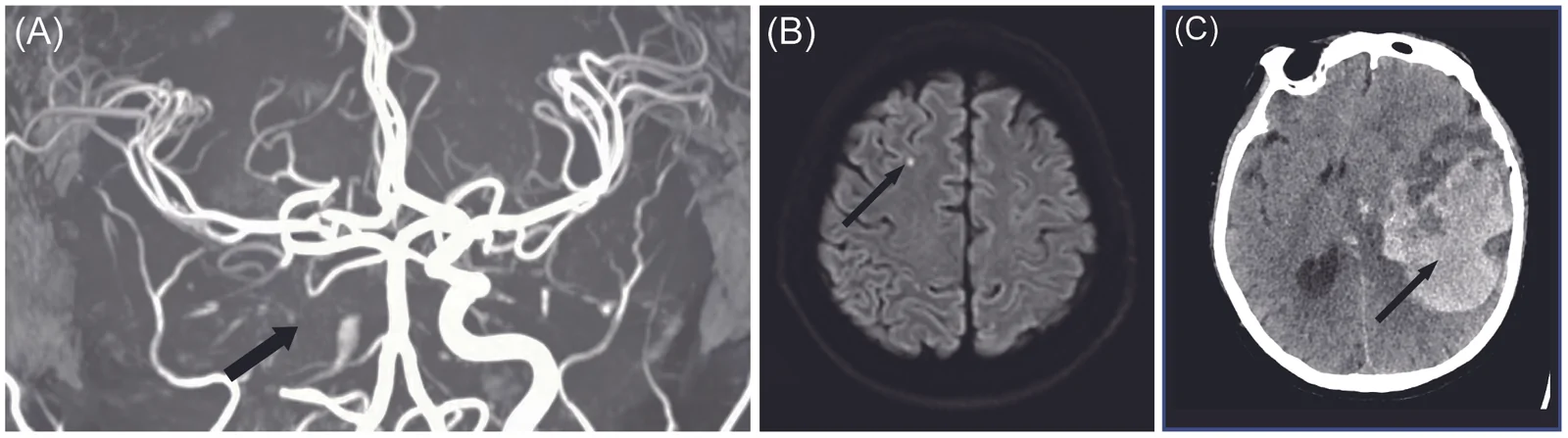
Representative neuroimaging findings corresponding to key components of the angioinvasion severity score. **(A)** Magnetic resonance angiography (MRA) showing occlusion of the internal carotid artery (arrow). **(B)** Diffusion-weighted imaging (DWI) demonstrating acute cerebral infarction (arrow). **(C)** Non-contrast computed tomography (CT) demonstrating intracranial hemorrhage (arrow).

Accordingly, moderate-to-severe angioinvasion scores were predominantly observed in ROCM (14/28, 50%) compared with RA (3/13, 23%) and CA (1/16, 6.3%) (*p* = 0.014), whereas absence of radiographic angioinvasion was most frequent in CA (13/16, 81%). Representative imaging findings corresponding to the components of the angioinvasion severity score are shown in **Figure 2**.

Other intracranial manifestations showed no significant differences among infection phenotypes, including meningeal involvement (14/28, 50% vs. 9/13, 69% vs. 9/16, 56%; *p* = 0.573), encephalitis-like lesions (12/28, 43% vs. 4/13, 31% vs. 7/16, 44%; *p* = 0.777), and intracranial hemorrhage (*p* = 0.763).

### 3.3 Treatment strategies and management patterns

Treatment patterns strongly reflected pathogen-specific antifungal selection and anatomical operability (**Table 3**). Combined antifungal-surgical management was performed in 20/28 (71%) ROCM, 12/13 (92%) RA, and 2/16 (13%) CA patients (*p* < 0.001). Conversely, antifungal therapy alone was predominant in CA (14/16, 88%).

**Table 3:** Treatment strategies, clinical outcomes, and complications across infection phenotypes.

Variable	N	Overall N = 57^1^	ROCM N = 28^1^	Rhino-cerebral Aspergillosis N = 13^1^	Cerebral Aspergillosis N = 16^1^	p-value^2^
Treatment modality						
Antifungal therapy alone	57	23 (40%)	8 (29%)	1 (7.7%)	14 (88%)	**<0.001**
Combined antifungal-surgical treatment	57	34 (60%)	20 (71%)	12 (92%)	2 (13%)	**<0.001**
Key antifungal exposure						
Any amphotericin B-based therapy	57	27 (47%)	25 (89%)	1 (7.7%)	1 (6.3%)	**<0.001**
Voriconazole-based therapy	57	32 (56%)	4 (14%)	13 (100%)	15 (94%)	**<0.001**
Isavuconazole-based therapy	57	25 (44%)	22 (79%)	1 (7.7%)	2 (13%)	**<0.001**
Combination antifungal therapy	57					**<0.001**
Monotherapy		31 (54%)	6 (21%)	12 (92%)	13 (81%)	
Dual therapy		26 (46%)	22 (79%)	1 (7.7%)	3 (19%)	
Surgical management						
Surgical intervention	57	34 (60%)	20 (71%)	12 (92%)	2 (13%)	**<0.001**
Treatment timing intervals						
Onset-to-antifungal interval (days)	57	21.0 (12.0-45.0)	15.0 (10.5-29.0)	63.0 (16.0-157.0)	28.5 (17.5-53.0)	**0.013**
Admission-to-antifungal interval (days)	57	4.0 (1.0-8.0)	3.0 (0.5-5.5)	4.0 (2.0-11.0)	6.0 (3.0-9.5)	0.147
Onset-to-surgery interval (days)	34	19.0 (10.0-40.0)	19.0 (10.5-29.5)	28.0 (12.0-118.5)	10.5 (7.0-14.0)	0.264
Admission-to-surgery interval (days)	34	4.0 (1.0-7.0)	5.0 (3.0-8.5)	2.0 (-14.5-5.0)	6.5 (6.0-7.0)	0.084
Total duration of antifungal therapy (days)	57	179.0 (77.0-382.0)	114.0 (22.0-424.5)	361.0 (202.0-408.0)	174.5 (156.5-238.5)	0.098
Mortality						
In-hospital mortality	57	9 (16%)	7 (25%)	1 (7.7%)	1 (6.3%)	0.278
30-day mortality	57	8 (14%)	7 (25%)	1 (7.7%)	0 (0%)	0.067
90-day mortality	57	14 (25%)	12 (43%)	1 (7.7%)	1 (6.3%)	**0.009**
Functional outcomes						0.092
mRS at 90 days	57					0.005
*0*		13 (23%)	2 (7.1%)	3 (23%)	8 (50%)	
*1*		18 (32%)	10 (36%)	5 (38%)	3 (19%)	
*2*		8 (14%)	2 (7.1%)	4 (31%)	2 (13%)	
*3*		0 (0%)	0 (0%)	0 (0%)	0 (0%)	
*4*		2 (3.5%)	1 (3.6%)	0 (0%)	1 (6.3%)	
*5*		2 (3.5%)	1 (3.6%)	0 (0%)	1 (6.3%)	
*6*		14 (25%)	12 (43%)	1 (7.7%)	1 (6.3%)	
Favorable/unfavorable outcome	57					**0.012**
*Favorable (mRS 0–2)*		39 (68%)	14 (50%)	12 (92%)	13 (81%)	
*unfavorable (mRS 3–6)*		18 (32%)	14 (50%)	1 (7.7%)	3 (19%)	
mRS at 180 days	57					**0.011**
*0*		14 (25%)	3 (11%)	3 (23%)	8 (50%)	
*1*		21 (37%)	10 (36%)	7 (54%)	4 (25%)	
*2*		4 (7.0%)	1 (3.6%)	2 (15%)	1 (6.3%)	
*3*		1 (1.8%)	0 (0%)	0 (0%)	1 (6.3%)	
*4*		1 (1.8%)	1 (3.6%)	0 (0%)	0 (0%)	
*5*		0 (0%)	0 (0%)	0 (0%)	0 (0%)	
*6*		16 (28%)	13 (46%)	1 (7.7%)	2 (13%)	
Favorable/unfavorable outcome	57					**0.012**
*Favorable (mRS 0-2)*		39 (68%)	14 (50%)	12 (92%)	13 (81%)	
*Unfavorable (mRS 3-6)*		18 (32%)	14 (50%)	1 (7.7%)	3 (19%)	
Follow-up duration (days)	57	247.0 (84.0-529.0)	106.5 (29.0-508.0)	515.0 (350.0-538.0)	186.0 (158.5-507.0)	**0.019**
Major complications						
Secondary cerebral infarction	57	23 (40%)	16 (57%)	5 (38%)	2 (13%)	**0.013**
Secondary intracranial hemorrhage	57	7 (12%)	4 (14%)	2 (15%)	1 (6.3%)	0.763
ICU admission	57	12 (21%)	11 (39%)	0 (0%)	1 (6.3%)	**0.003**
Mechanical ventilation	57	4 (7.0%)	4 (14%)	0 (0%)	0 (0%)	0.172
Treatment-related adverse events						
Renal toxicity	50	17 (34%)	12 (57%)	2 (15%)	3 (19%)	**0.017**
Hepatic toxicity	51	1 (2.0%)	0 (0%)	1 (7.7%)	0 (0%)	0.255
Electrolyte disturbance	50	18 (36%)	11 (52%)	5 (38%)	2 (13%)	**0.045**

^1^n (%); Median (Q1-Q3).

^2^Fisher’s exact test; Kruskal-Wallis rank sum test.

ICU, intensive care unit; mRS, modified Rankin Scale.

Bold values indicate statistically significant differences (p < 0.05).

ROCM was primarily treated with amphotericin B- and isavuconazole-based regimens, frequently in combination, whereas voriconazole-based therapy predominated in aspergillosis (all *p* < 0.001). Surgical intervention was substantially more common in ROCM and RA than in CA (71% vs. 92% vs. 13%, *p* < 0.001), likely reflecting differences in anatomical accessibility.

Treatment timing also differed across groups. RA demonstrated the longest onset-to-antifungal interval (median 63 days), whereas ROCM showed the shortest interval (median 15 days; *p* = 0.013). No significant between-group differences were observed for overall antifungal treatment duration or surgical timing variables.

### 3.4 Clinical outcomes across infection phenotypes

Clinical outcomes differed substantially across infection phenotypes (**Table 3**). ROCM demonstrated the highest 90-day mortality (12/28, 43%) compared with RA (1/13, 7.7%) and CA (1/13, 6.3%) (*p* = 0.009).

**Figure 3:**
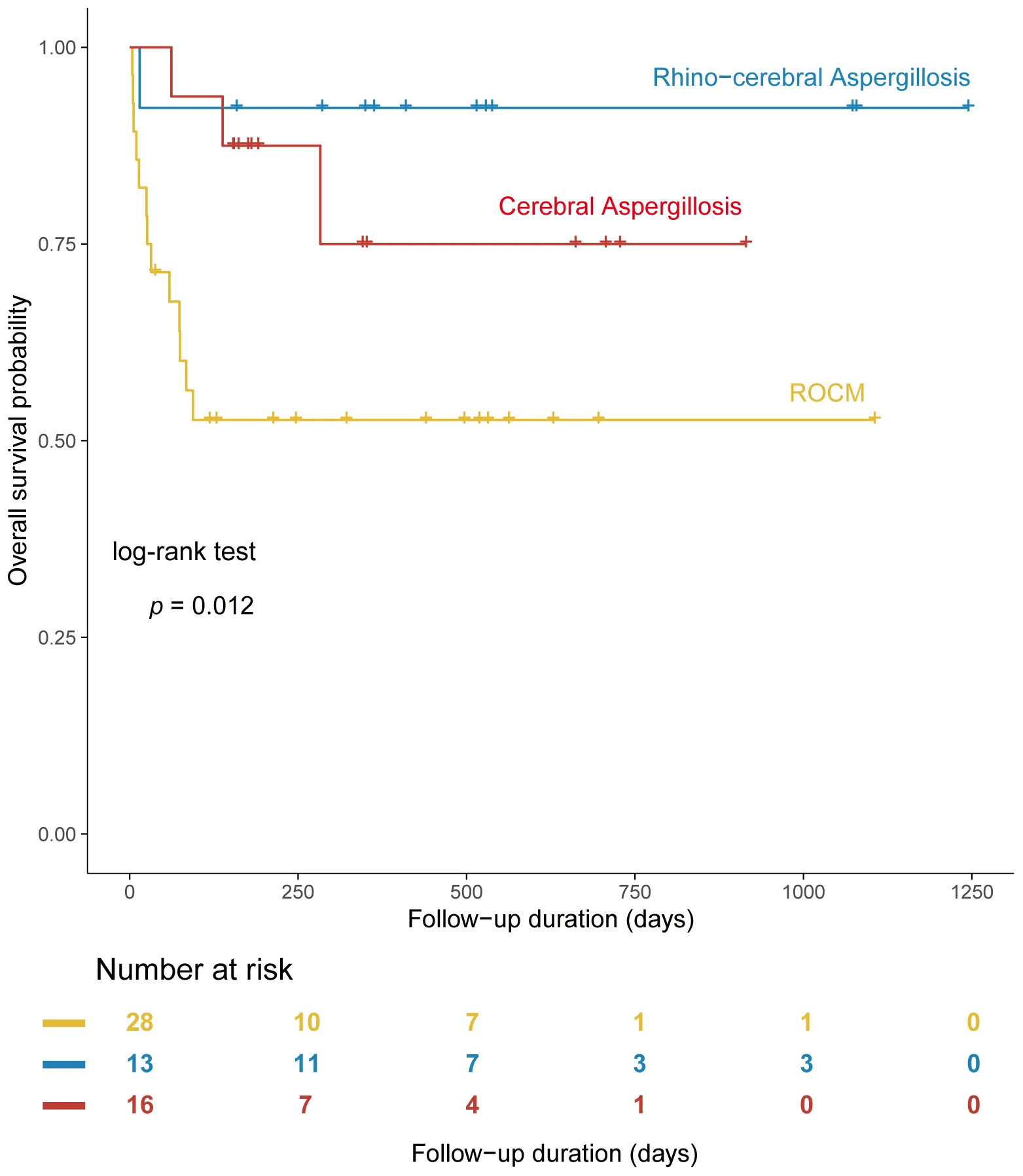
Kaplan–Meier survival curves stratified by infection phenotype. Kaplan–Meier survival analysis demonstrating overall survival among patients with ROCM, rhino-cerebral aspergillosis, and cerebral aspergillosis during follow-up. Survival differences among groups were evaluated using the log-rank test.

Across the entire follow-up period, Kaplan–Meier analysis showed significant survival differences among infection phenotypes (**Figure 3**). ROCM exhibited the poorest survival profile with earlier and more frequent mortality events, whereas RA showed the most favorable survival pattern (log-rank *p* = 0.01).

Neurological functional outcomes at 90 days similarly differed across groups. Favorable 90-day functional outcome (mRS 0–2) was achieved in 68% of the cohort overall, but was substantially less frequent in ROCM (14/18, 50%) than in RA (12/13, 92%) and CA (13/16, 81%) (*p* = 0.012).

This difference in functional outcomes remained significant at 180 days. The full 180-day mRS distribution differed significantly across infection phenotypes (*p* = 0.011), and favorable 180-day functional outcome (mRS 0–2) was achieved in 14/28 (50%) ROCM, 12/13 (92%) RA, and 13/16 (81%) CA patients (*p* = 0.012).

Neurovascular complications and intensive care requirements differed among infection phenotypes. Secondary cerebral infarction occurred more frequently in ROCM (16/28, 57%) than in RA (5/13, 38%) and CA (2/16, 13%) (*p* = 0.013). ICU admission was also more frequent in ROCM (11/28, 39%) compared with RA (0/13, 0%) and CA (1/16, 6.3%) (*p* = 0.003). In contrast, secondary intracranial hemorrhage (4/28, 14% vs. 2/13, 15% vs. 1/16, 6.3%; *p* = 0.763) and mechanical ventilation (4/28, 14% vs. 0/13, 0% vs. 0/16, 0%; *p* = 0.172) were not significantly different among groups.

Treatment-related adverse events varied among groups. Renal toxicity occurred more frequently in ROCM (12/21, 57%) than RA (2/13, 15%) and CA (3/16, 19%) (*p* = 0.017). Electrolyte disturbances were also more common in ROCM (11/21, 52% vs. 5/13, 38% vs. 2/16, 13%; *p* = 0.045). Hepatic toxicity was uncommon and did not differ significantly among groups (0 vs. 1/13, 7.7% vs. 0; *p* = 0.255).

### 3.5 Predictors of all-cause mortality during follow-up

Univariable Cox regression analysis identified several factors associated with mortality during follow-up (**Table 4**). Compared with ROCM, RA was associated with lower mortality risk (HR 0.12, 95% CI 0.02–0.92; *p* = 0.041), whereas CA showed a non-significant trend toward lower mortality (HR 0.30, 95% CI 0.08–1.05; *p* = 0.059). Higher admission GCS score was associated with reduced mortality risk (HR 0.75, 95% CI 0.66–0.84; *p* < 0.001), and higher lymphocyte count was also associated with lower mortality risk (HR 0.27, 95% CI 0.09–0.81; *p* = 0.019).

**Table 4:** Univariable and multivariable Cox regression analyses for mortality.

Variable	Univariable HR (95% CI)	Univariable P value	Multivariable HR (95% CI)	Multivariable P value
Infection group				
ROCM	Reference	—	Reference	—
Rhino-cerebral aspergillosis	0.12 (0.02-0.92)	0.041	0.18 (0.02-1.57)	0.122
erebral aspergillosis	0.30 (0.08-1.05)	0.059	0.39 (0.10-1.53)	0.178
Age	1.03 (0.99-1.07)	0.157	1.05 (1.00-1.10)	0.034
Admission GCS score	0.75 (0.66-0.84)	<0.001	0.77 (0.67-0.88)	<0.001
Lymphocyte count	0.27 (0.09-0.81)	0.019	0.45 (0.15-1.34)	0.150

HR, hazard ratio; CI, confidence interval; ROCM, rhino-orbital-cerebral mucormycosis; GCS, Glasgow Coma Scale.

**Figure 4:**
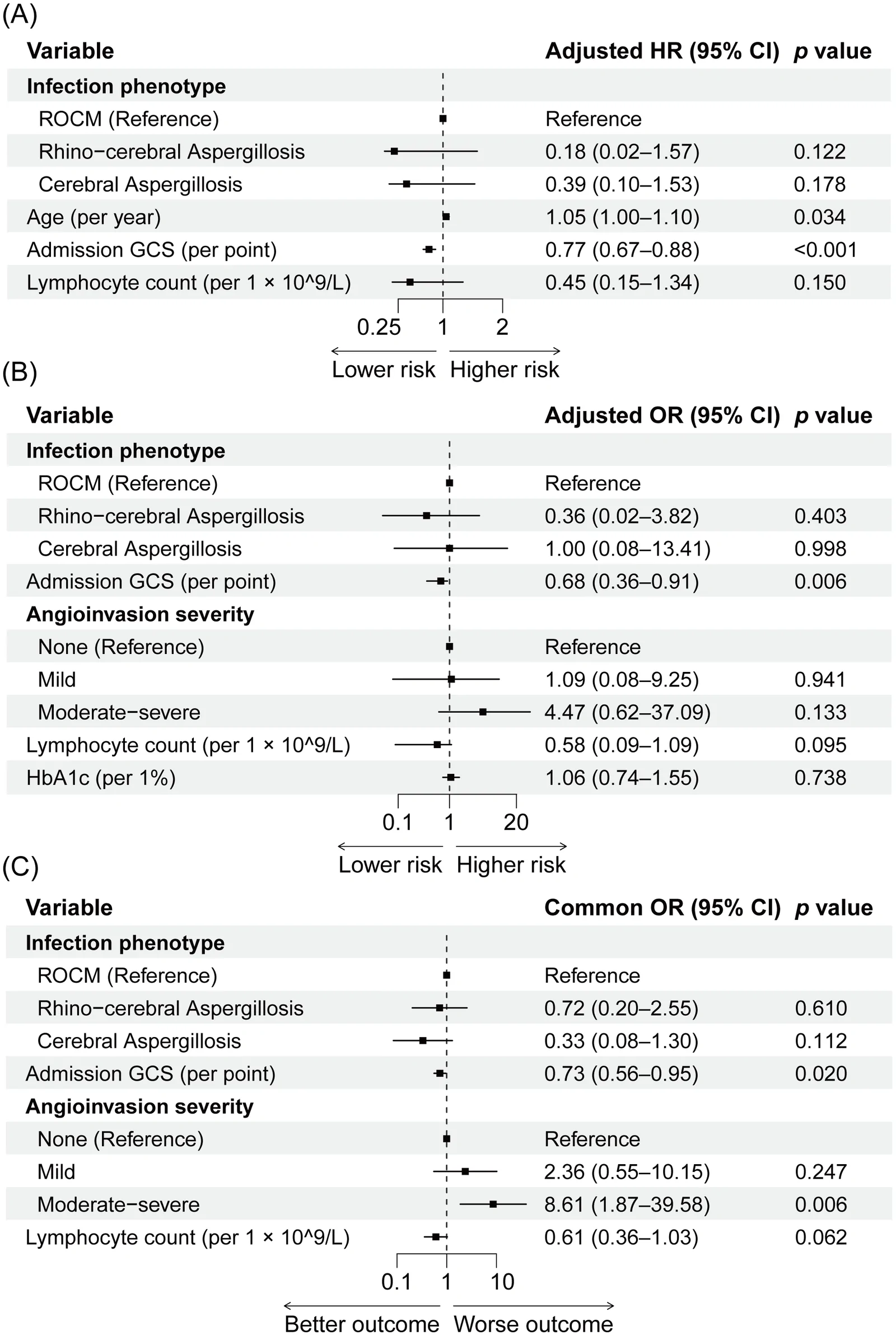
Multivariable regression analyses for mortality and neurological functional outcomes. **(A)** Forest plot showing adjusted HRs and 95% CIs for factors associated with mortality during follow-up derived from the multivariable Cox proportional hazards model. HR >1 indicates increased mortality risk. **(B)** Forest plot showing adjusted ORs and 95% CIs for predictors of unfavorable functional outcome (mRS score ≥3) derived from the multivariable Firth penalized logistic regression model, which was used to reduce small-sample bias and address potential separation issues. OR >1 indicates higher odds of unfavorable functional outcome. **(C)** Forest plot showing common ORs and 95% CIs derived from the proportional odds ordinal logistic regression model using the full 90-day mRS distribution (0–6). Common OR >1 indicates a shift toward worse neurological disability across the entire mRS spectrum.

In multivariable Cox regression analysis, higher admission GCS score remained independently associated with reduced mortality (adjusted HR 0.77, 95% CI 0.67–0.88; *p* < 0.001) (**Figure 4A**). Older age was independently associated with increased mortality risk (adjusted HR 1.05, 95% CI 1.00–1.10; *p* = 0.034). In contrast, infection phenotype (RA vs ROCM: adjusted HR 0.18, 95% CI 0.02–1.57; *p* = 0.122; CA vs ROCM: adjusted HR 0.39, 95% CI 0.10–1.53; *p* = 0.178) and lymphocyte count (adjusted HR 0.45, 95% CI 0.15–1.34; *p* = 0.150) were not independently associated with mortality after adjustment.

Restricted cubic spline analysis further demonstrated a significant nonlinear association between admission GCS score and mortality risk (*p* < 0.001), with sharply increased mortality observed at lower GCS levels (**Supplementary Figure 1A**). In contrast, no significant nonlinear relationship was observed between lymphocyte count and mortality risk (**Supplementary Figure 1B**).

### 3.6 Predictors of unfavorable functional outcome at 90 and 180 days

Baseline characteristics stratified according to 90-day functional outcome are summarized in **Supplementary Table 3**. Overall, 18 patients (32%) experienced unfavorable outcome (mRS 3–6). Patients with unfavorable outcome demonstrated more severe neurological impairment and a greater burden of angioinvasive disease, including lower admission GCS scores, consciousness disturbance, hemiplegia, cerebral infarction, ICA involvement, cavernous sinus involvement, and moderate-to-severe angioinvasion. Higher HbA1c and inflammatory marker levels were also observed among patients with poor outcome.

Univariable Firth logistic regression demonstrated that higher admission GCS score was associated with lower odds of unfavorable 90-day functional outcome (OR 0.60, 95% CI 0.42–0.77; *p* < 0.001) (**Table 5**). Moderate-to-severe angioinvasion was strongly associated with unfavorable outcome compared with no angioinvasion (OR 12.38, 95% CI 3.08–62.17; *p* < 0.001). Compared with ROCM, RA was associated with lower odds of unfavorable outcome (OR 0.14, 95% CI 0.01–0.73; *p* = 0.018), whereas CA showed a non-significant trend toward lower odds (OR 0.28, 95% CI 0.06–1.07; *p* = 0.062).

**Table 5:** Univariable and multivariable Firth logistic regression analyses for 90-day unfavorable functional outcome.

Variable	Univariable OR (95% CI)	Univariable P value	Multivariable OR (95% CI)	Multivariable P value
Infection group				
ROCM	Reference	—	Reference	—
Rhino-cerebral aspergillosis	0.14 (0.01–0.73)	0.018	0.36 (0.02–3.82)	0.403
Cerebral Aspergillosis	0.28 (0.06–1.07)	0.062	1.00 (0.08–13.41)	0.998
Admission GCS	0.60 (0.42–0.77)	<0.001	0.68 (0.36–0.91)	0.006
Angioinvasion severity				
None	Reference	—	Reference	—
Mild angioinvasion	2.33 (0.33–14.61)	0.370	1.09 (0.08–9.25)	0.941
Moderate-severe angioinvasion	12.38 (3.08–62.17)	<0.001	4.47 (0.62–37.09)	0.133
Lymphocyte count	0.34 (0.09–1.04)	0.065	0.58 (0.09–1.09)	0.095
HbA1c	1.31 (1.06–1.67)	0.011	1.06 (0.74–1.55)	0.738

OR, odds ratio; CI, confidence interval; ROCM, rhino-orbital-cerebral mucormycosis; GCS, Glasgow Coma Scale.

In multivariable Firth logistic regression, higher admission GCS score remained independently associated with lower odds of unfavorable functional outcome (adjusted OR 0.68, 95% CI 0.36–0.91; *p* = 0.006) (**Figure 4B**). Moderate-to-severe angioinvasion retained an adverse association but did not reach statistical significance after adjustment (adjusted OR 4.47, 95% CI 0.62–37.09; *p* = 0.133). Higher lymphocyte count (adjusted OR 0.58, 95% CI 0.09–1.09; *p* = 0.095) and HbA1c (adjusted OR 1.06, 95% CI 0.74–1.55; *p* = 0.738) were not independently associated with unfavorable outcome. Infection phenotype was also not independently associated with unfavorable outcome after adjustment (RA vs ROCM: adjusted OR 0.36, 95% CI 0.02–3.82; *p* = 0.403; CA vs ROCM: adjusted OR 1.00, 95% CI 0.08–13.41; *p* = 0.998).

**Figure 5:**
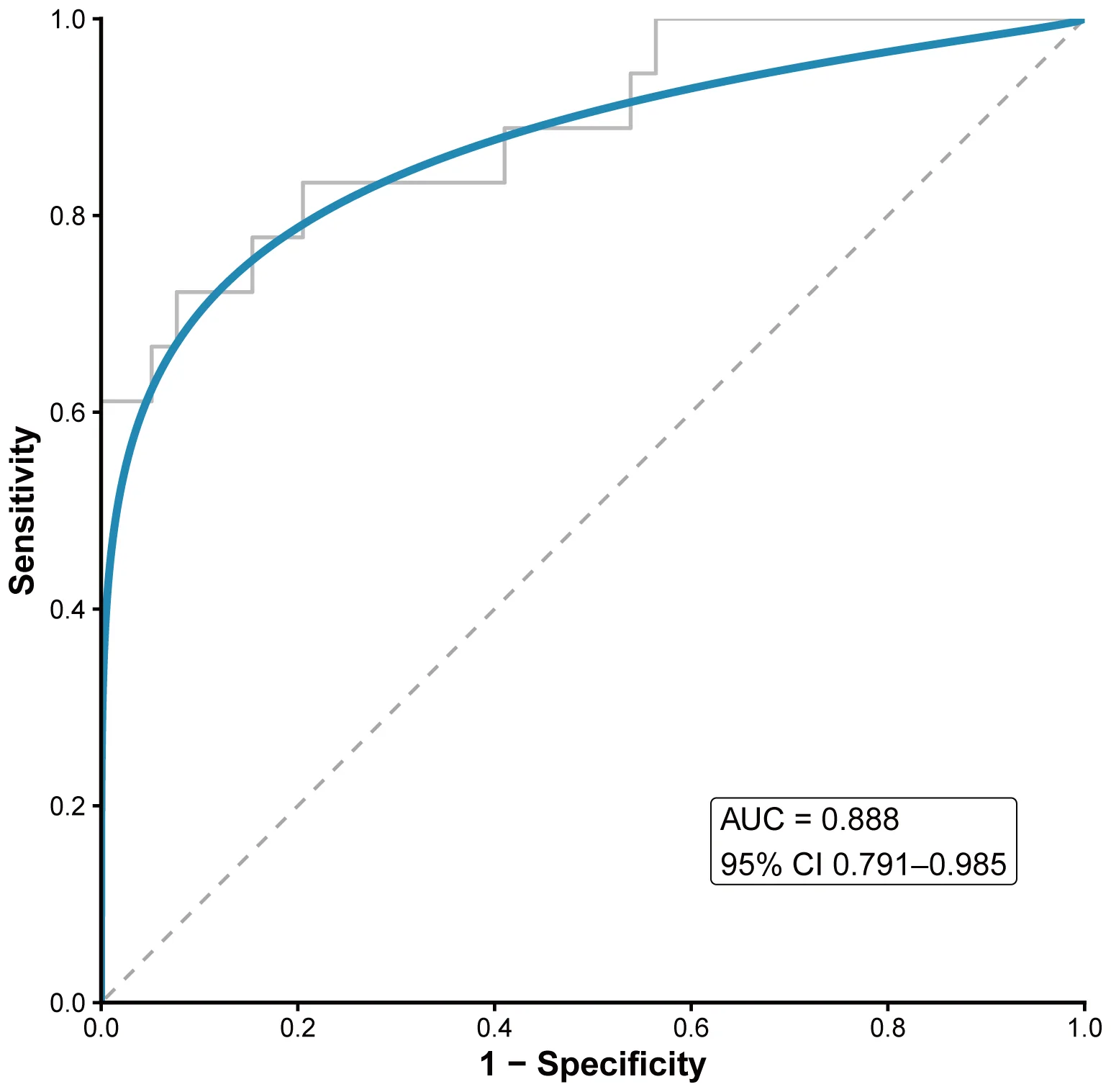
Receiver operating characteristic curve for prediction of unfavorable 90-day functional outcome. ROC curve demonstrating the discriminative performance of the multivariable Firth logistic regression model for predicting unfavorable functional outcome (90-day mRS ≥3). The area under the curve (AUC) with corresponding 95% confidence interval is shown.

The multivariable prediction model demonstrated good discriminative performance for unfavorable functional outcome, with an AUC of 0.888 (95% CI 0.791–0.985) (**Figure 5**).

Additional analyses of 180-day unfavorable functional outcome showed highly consistent findings (**Supplementary Figure 2**). In multivariable Firth penalized logistic regression, higher admission GCS score remained independently associated with lower odds of unfavorable 180-day functional outcome (adjusted OR 0.68, 95% CI 0.36–0.91; *p* = 0.006). Moderate-to-severe angioinvasion showed a persistent adverse association but did not reach statistical significance after adjustment (adjusted OR 4.47, 95% CI 0.62–37.09; *p* = 0.133). Higher lymphocyte count demonstrated a trend toward lower odds of unfavorable outcome but was not statistically significant (adjusted OR 0.58, 95% CI 0.09–1.09; *p* = 0.095). HbA1c was not associated with 180-day unfavorable outcome (adjusted OR 1.06, 95% CI 0.74–1.55; *p* = 0.738). Infection phenotype was also not independently associated with outcome (RA vs ROCM: adjusted OR 0.36, 95% CI 0.02–3.82; *p* = 0.403; CA vs ROCM: adjusted OR 1.00, 95% CI 0.08–13.41; *p* = 0.998).

### 3.7 Ordinal shift analysis of 90-day and 180-day mRS outcomes

**Figure 6:**
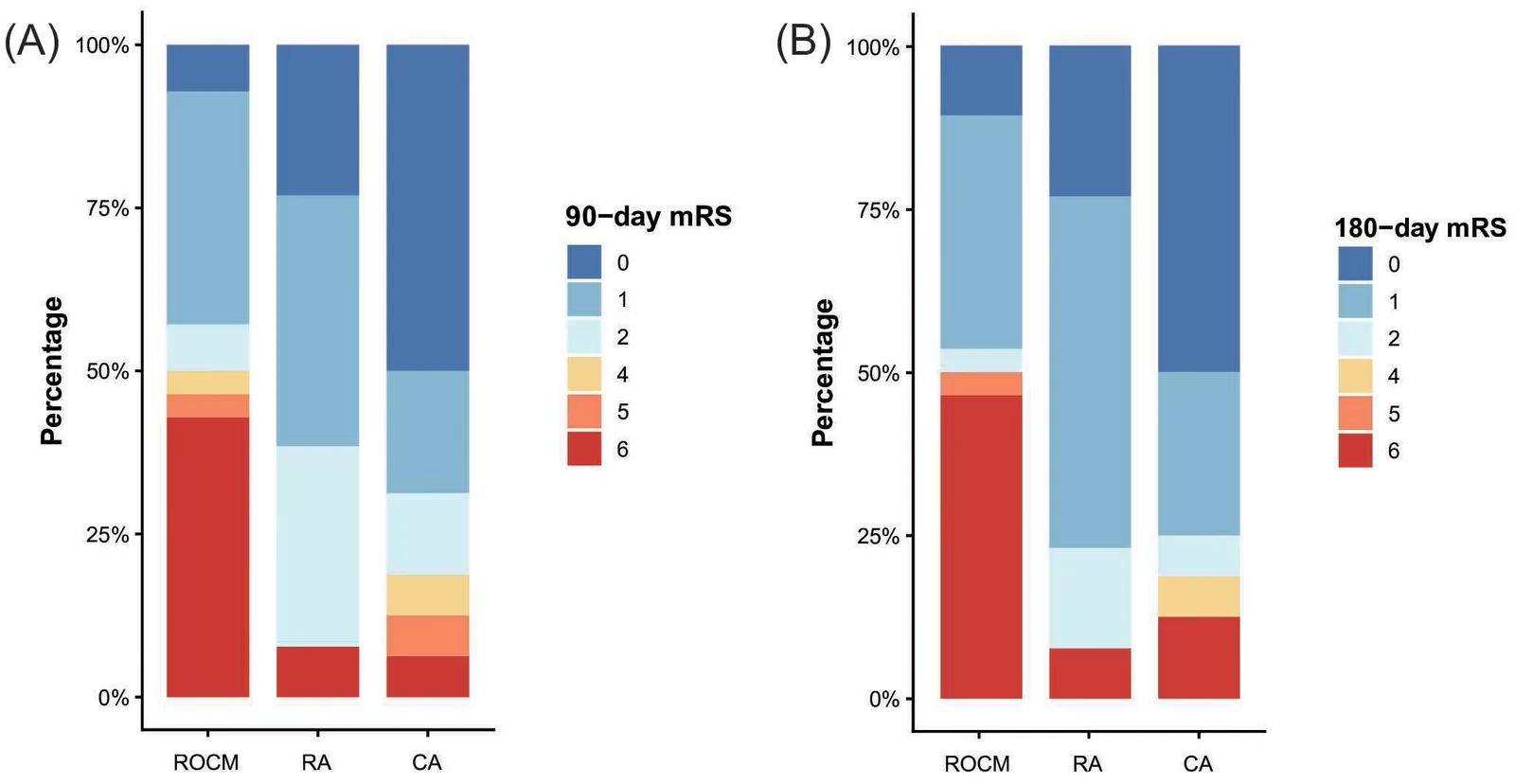
Distribution of 90-day and 180-day mRS scores according to infection phenotype. **(A)** Stacked bar plot showing the full distribution of 90-day mRS scores across the three infection phenotypes: ROCM, RA, and CA. **(B)** Stacked bar plot showing the full distribution of 180-day mRS scores across the same infection phenotypes.

Ordinal shift analysis using proportional odds logistic regression was performed to evaluate the full distribution of 90-day mRS outcomes across the disability spectrum (**Supplementary Table 4**, **Figures 6A**, **4C**). Patients with ROCM exhibited higher rates of severe disability and mortality, while those with RA and CA demonstrated a relatively more favorable distribution of neurological outcomes.

In the 90-day ordinal logistic regression model, lower admission GCS score was independently associated with a shift toward worse functional outcomes across the entire mRS distribution (common OR 0.73, 95% CI 0.56–0.95; *p* = 0.020). Moderate-to-severe angioinvasion was also strongly associated with worse ordinal disability outcomes compared with no angioinvasion (common OR 8.61, 95% CI 1.87–39.58; *p* = 0.006). Lower lymphocyte count demonstrated a non-significant trend toward poorer functional outcomes, whereas infection phenotype was not independently associated with ordinal mRS shift after adjustment.

Additional ordinal shift analysis at 180 days demonstrated consistent associations across the full mRS distribution (**Figure 6B**, **Supplementary Figure 2**). Higher admission GCS score was associated with a shift toward better functional outcomes (common OR 0.74, 95% CI 0.56–0.97; *p* = 0.028). Moderate-to-severe angioinvasion remained independently associated with a shift toward worse functional outcomes (common OR 7.10, 95% CI 1.48–34.13; *p* = 0.014). Infection phenotype and lymphocyte count were not independently associated with 180-day ordinal mRS shift.

Overall, the 90-day and 180-day ordinal shift analysis consistently demonstrated that admission neurological severity and angioinvasion burden, rather than infection phenotype alone, were the principal determinants of worse disability across the mRS spectrum.

## 4 Discussion

In this comparative cohort of invasive rhino-cerebral and cerebral fungal infections, we investigated the relationship between fungal phenotype, angioinvasive neurovascular injury, and clinical outcomes. Several important findings emerged. First, a moderate-to-severe angioinvasion severity score (≥2) was independently associated with a shift toward worse 90-day neurological functional outcomes across the ordinal mRS distribution (common OR 8.61, 95% CI 1.87–39.58). Second, admission GCS score was the strongest independent predictor of both mortality and unfavorable functional outcome. Third, the three infection phenotypes—ROCM, RA, and CA—exhibited distinct angioinvasion patterns, with ROCM predominantly showing large-vessel involvement and CA displaying hematogenous dissemination with abscess formation. Fourth, host immune status played a key role in susceptibility: patients with ROCM were characterized by uncontrolled diabetes and diabetic ketoacidosis, whereas those with CA predominantly presented with lymphopenia and hematological malignancies. These findings transform angioinvasion from a qualitative pathological feature into a quantifiable prognostic marker, providing a new tool for clinical risk stratification and treatment decision-making.

### 4.1 Angioinvasion as the central mechanism driving poor outcomes

This study further suggests that fungal angioinvasion and the resulting angioinvasion burden angioinvasion may represent a central mechanism underlying poor outcomes in invasive CNS fungal infection. Fungal angioinvasion causes endothelial destruction, thrombosis, vascular occlusion, hemorrhage, and subsequent ischemic-necrotic brain injury (Chantelot et al., 2026; Kontoyiannis and Walsh, 2026). The spore coat protein homolog CotH3 on Mucorales surfaces binds to glucose-regulated protein 78 (GRP78) on host endothelial cells, initiating vascular invasion (Soliman et al., 2021). ROCM appears particularly prone to contiguous skull-base vascular invasion because of its anatomical proximity to major intracranial vessels and cranial neurovascular structures (Lersy et al., 2022; La Porte et al., 2026). Infection may extend from the paranasal sinuses into the orbit and adjacent facial soft tissues, subsequently spreading through the orbital apex and superior orbital fissure to involve the cavernous sinus and ICA (Hosseini and Borghei, 2005; Anders et al., 2015). A recent case series highlighted that ICA thrombosis is a critical complication of angioinvasive mucormycosis, often predicting subsequent cerebral infarction and poor prognosis (Surbhi et al., 2025).

Consistent with these mechanisms, our cohort revealed distinct neuroimaging patterns of vascular involvement and intracranial dissemination across infection phenotypes. ROCM (n=28) was characterized by contiguous sinonasal-skull base extension with a higher burden of angioinvasion-related imaging findings—ICA involvement in 50%, cerebral infarction in 57%, and moderate-to-severe angioinvasion score in 50% of cases. These findings are broadly consistent with recent observations from the MAGICMUCOR study, which highlighted heterogeneous patterns of intracranial extension in ROCM depending on the presence of bone involvement and other anatomical pathways (La Porte et al., 2026). RA (n=13) showed an intermediate imaging phenotype, with less frequent but still substantial vascular involvement. The prolonged median onset-to-antifungal interval (63 days) may reflect the diagnostic challenges associated with CNS aspergillosis, which often presents with nonspecific early manifestations (Danion et al., 2019; Leroy et al., 2020). A systematic review of 235 CNS aspergillosis cases reported neuroimaging findings including cerebral abscess (70.2%), meningitis (14%), infarction (13.2%), and mycotic aneurysm (8.9%) (Meena et al., 2021), consistent with our observations. CA (n=16) predominantly showed imaging features suggestive of hematogenous dissemination, including brain abscess formation and multifocal intracranial lesions, with less frequent radiological manifestations of major vessel involvement, consistent with the CEREALS national cohort study (Serris et al., 2022).

Although ROCM demonstrated the worst crude outcomes, infection phenotype itself was not independently associated with prognosis after multivariable adjustment. Instead, neurological severity and angioinvasion burden consistently emerged as the dominant determinants of mortality and disability. These findings suggest that the poorer prognosis traditionally associated with ROCM may be partly explained by its higher frequency of radiological manifestations of angioinvasion and neurovascular complications rather than by fungal subtype alone (Gouzien et al., 2024). Importantly, ordinal shift analyses demonstrated that greater angioinvasion burden, as quantified by the angioinvasion severity score, was independently associated with worse neurological disability outcomes across the mRS spectrum, whereas multivariable logistic regression showed a consistent but attenuated adverse association. This distinction shifts prognostic interpretation from pathogen-based classification toward mechanism-based risk stratification centered on radiological evidence of vascular involvement and early neurological impairment.

### 4.2 Neurological severity as an integrated clinical marker of angioinvasion burden

Lower admission GCS score was an independent predictor of both mortality and unfavorable neurological outcome across Cox, Firth logistic, and ordinal shift models, highlighting its robustness as an early prognostic marker in invasive CNS fungal infection (Meena et al., 2021; Zia et al., 2025). Reduced GCS likely reflects high angioinvasion burden caused by fungal angioinvasion, including infarction, hemorrhage, and diffuse cerebral dysfunction (Chikley et al., 2019; Chantelot et al., 2026). ICA thrombosis can compromise hemispheric perfusion, leading to large territorial infarctions that directly reduce consciousness levels; multifocal infarcts involving bilateral hemispheres or brainstem structures compound this effect, while secondary cerebral edema further worsens neurological status (Kashyap et al., 2019; Little et al., 2019). In cryptococcal meningitis, higher GCS scores at presentation have similarly been shown to predict favorable outcomes, reinforcing GCS as a broadly applicable prognostic indicator in CNS fungal infections (Cornely et al., 2019). Notably, although GCS may have limitations—dominant hemisphere infarcts causing aphasia with preserved motor responses can yield normal GCS scores, and isolated brainstem lesions may produce depressed consciousness without typical hemispheric signs—GCS remained the strongest independent prognostic factor in our multivariable analyses. Importantly, restricted cubic spline analysis demonstrated a significant nonlinear association between GCS and mortality, with risk increasing disproportionately at lower GCS levels, suggesting that severe impairment of consciousness may represent a critical threshold of irreversible neurological injury.

### 4.3 Host susceptibility: glycemic status, lymphopenia, and immune state

Our study revealed two distinct host susceptibility patterns. Patients with ROCM were predominantly characterized by uncontrolled diabetes mellitus (93%) and diabetic ketoacidosis (36%), with markedly higher HbA1c and glucose levels compared with the other groups. In contrast, CA patients in our cohort showed a higher prevalence of classical immunocompromising conditions, including hematological malignancy and immunosuppressive therapy, although these differences did not reach statistical significance. These findings may reflect the underlying host susceptibility patterns associated with CNS aspergillosis; however, the limited sample size precludes definitive conclusions regarding phenotype-specific risk factors.

The molecular mechanisms underlying this dichotomy can be summarized as follows. Hyperglycaemia and ketoacidosis upregulate endothelial GRP78 expression, providing more receptors for the Mucorales CotH3-GRP78 interaction and thereby facilitating fungal angioinvasion (Alqarihi et al., 2020). In addition, hyperglycaemia impairs neutrophil chemotaxis and phagocytic function (Abdolalizadeh et al., 2020). Conversely, lymphopenia primarily reflects a T-cell immunodeficiency state and is most frequently observed in patients with hematological malignancies receiving chemotherapy or undergoing hematopoietic stem cell transplantation (Rammaert et al., 2012; Latgé and Chamilos, 2019). T cells play a central role in anti-Aspergillus immunity by secreting cytokines such as IFN-γ, which activate macrophages and neutrophils (Cramer et al., 2011). Studies have demonstrated that lymphopenic patients exhibit significantly impaired anti-Aspergillus immune responses and are more susceptible to haematogenous dissemination (Susianti et al., 2021). These distinct susceptibility patterns underscore the importance of phenotype-specific management, which is further discussed in the clinical implications section below.

### 4.4 Clinical implications for risk stratification and management

A major clinical implication of our findings is that early recognition of fungal neurovascular involvement may improve risk stratification and management. Given the strong association between angioinvasion burden and poor neurological outcomes, vascular-focused neuroimaging assessment—including evaluation for ICA involvement, cerebral infarction, cavernous sinus invasion, and intracranial hemorrhage—should be emphasized in suspected invasive CNS fungal infection. A recent review highlighted that timely recognition of neuroimaging findings, such as the “black turbinate sign” and lack of contrast enhancement, can aid neuroradiologists in distinguishing angioinvasive fungal infections and improving patient outcomes (Safder et al., 2010; Kassotis et al., 2025). Patients with extensive angioinvasive disease may require more aggressive monitoring, earlier multidisciplinary intervention, intensive antifungal therapy, and timely surgical consideration when feasible (Cornely et al., 2019; Kontoyiannis and Walsh, 2026). For patients with moderate-to-severe angioinvasion, higher doses of liposomal amphotericin B (≥7.5 mg/kg/day) should be considered, as guidelines recommend up to 10 mg/kg/day for CNS involvement (Cornely et al., 2019), and treatment delay beyond 6 days from symptom onset has been shown to double mortality (Chamilos et al., 2008). However, recipients of increased doses tended to have increased response rates (Lanternier et al., 2015).

In our cohort, antifungal treatment patterns closely followed international recommendations. ROCM predominantly received amphotericin B-based regimens with or without isavuconazole, whereas aspergillosis was primarily treated with voriconazole—a first-line agent for CNS aspergillosis due to its favorable CNS penetration. When cultures remain negative, molecular diagnostics such as metagenomic next-generation sequencing can aid pathogen identification and guide antifungal therapy (Wei et al., 2024). Surgical intervention rates were substantially higher in ROCM and RA (71% and 92%, respectively) than in CA (13%), reflecting differences in anatomical accessibility. Debridement until healthy, bleeding tissue is critical for local disease control (Sweed et al., 2025), and multiple studies have confirmed that combined surgical and antifungal therapy significantly improves survival; even in severe ROCM, this approach may provide survival benefit (Roxbury et al., 2017; Soare et al., 2020). Therefore, for patients with moderate-to-severe angioinvasion, multidisciplinary early surgical debridement should be prioritized (Cornely et al., 2019).

Notably, outcome assessment should extend beyond survival alone, as substantial neurological disability remained common among survivors with severe neurovascular injury. The consistency between binary and ordinal analyses further supports the value of evaluating the full mRS distribution to better capture overall disease burden and treatment effect. Beyond pathogen-directed therapy, our observation of distinct host susceptibility patterns—marked hyperglycemia in ROCM versus lymphopenia in cerebral aspergillosis—underscores the need for phenotype-specific adjunctive management. For ROCM patients with uncontrolled diabetes or diabetic ketoacidosis, aggressive metabolic correction is essential (Alqarihi et al., 2020; Liang et al., 2024). For lymphopenic patients with CNS aspergillosis, immune reconstitution is paramount, and corticosteroid exposure should be rapidly tapered to the lowest effective dose (Pagano et al., 2011; Li and Denning, 2023). Collectively, optimal management of invasive CNS fungal infection requires an integrated approach that combines timely antifungal therapy, early surgical debridement, and aggressive reversal of underlying host immune and metabolic abnormalities tailored to each patient’s infection phenotype.

### 4.5 Study limitations

This study has several limitations.

First, the retrospective design introduces inherent selection bias; however, the use of a centralized imaging review process partially mitigated this issue. Second, because this was a retrospective study of a rare disease, we included all consecutive eligible cases during the study period. Nevertheless, the sample size (n=57) was modest, particularly for subgroup analyses in which some outcome events were few, and this may have limited statistical precision. Therefore, the present findings should be interpreted as hypothesis-generating and require confirmation in larger multicenter cohorts. The observed event rates and effect estimates may also help inform sample-size planning for future prospective studies. Third, the angioinvasion severity score is based on radiographic interpretation, which carries interobserver variability, although independent assessment by two neuroradiologists with consensus agreement helped control this variation. Fourth, unmeasured confounders—including fungal burden, antifungal drug serum concentrations, species-level resistance profiles, and detailed inflammatory marker kinetics—could not be fully excluded. Fifth, treatment decisions were made by clinicians based on individual patient conditions rather than by a randomized protocol, which may have introduced indication bias and limited causal inference. Sixth, the single-country and primarily tertiary-hospital setting may limit generalizability to other healthcare systems or less specialized centers. Seventh, we did not systematically evaluate serial changes in angioinvasion scores before and after treatment, precluding assessment of whether the score could serve as a dynamic marker of therapeutic response. In addition, although the angioinvasion severity score showed biological plausibility and internal prognostic relevance in this cohort, it remains an exploratory imaging-based tool and has not yet undergone formal external validation; therefore, its reproducibility and calibration require confirmation in prospective multicenter studies.

This study provides the first systematic quantification of angioinvasion severity in invasive CNS fungal infection and demonstrates its independent association with worse ordinal neurological functional outcome. The angioinvasion severity score may help identify patients who could benefit from more aggressive therapeutic strategies, including higher-dose liposomal amphotericin B and earlier neurosurgical consultation. Admission GCS independently predicted both mortality and unfavorable functional outcome, serving as an accessible clinical indicator of early neurological severity. Future research should focus on prospective multi-center validation of this scoring system and integration with novel biomarkers to improve risk stratification and guide individualized treatment in these devastating infections.

## Data Availability

The raw data supporting the conclusions of this article will be made available by the authors, without undue reservation.

## References

[B1] Abdolalizadeh P. Kashkouli M. B. Khademi B. Karimi N. Hamami P. Es'haghi A. (2020). Diabetic versus non-diabetic rhino-orbito-cerebral mucormycosis. Mycoses 63, 573–578. doi: 10.1111/myc.13078 32191363

[B2] Alqarihi A. Gebremariam T. Gu Y. Swidergall M. Alkhazraji S. Soliman S. S. M. . (2020). GRP78 and integrins play different roles in host cell invasion during mucormycosis. mBio 11, e01087-20. doi: 10.1128/mBio.01087-20 32487760 PMC7267888

[B3] Anders U. M. Taylor E. J. Martel J. R. Martel J. B. (2015). Acute orbital apex syndrome and rhino-orbito-cerebral mucormycosis. Int. Med. Case Rep. J. 8, 93–96. doi: 10.2147/IMCRJ.S83036 25945068 PMC4407820

[B4] Bongomin F. Gago S. Oladele R. O. Denning D. W. (2017). Global and multi-national prevalence of fungal diseases-estimate precision. J. Fungi (Basel) 3, 57. doi: 10.3390/jof3040057 29371573 PMC5753159

[B5] Chamilos G. Lewis R. E. Kontoyiannis D. P. (2008). Delaying amphotericin B-based frontline therapy significantly increases mortality among patients with hematologic Malignancy who have zygomycosis. Clin. Infect. Dis. 47, 503–509. doi: 10.1086/590004 18611163

[B6] Chantelot L. Sitterlé E. Poirée S. Jullien V. Danion F. Serris A. (2026). Cerebral aspergillosis: diagnostic challenges, therapeutic strategies, and future research. CNS Drugs 40, 43–58. doi: 10.1007/s40263-025-01241-0 41214263 PMC12769985

[B7] Chikley A. Ben-Ami R. Kontoyiannis D. P. (2019). Mucormycosis of the central nervous system. J. Fungi (Basel) 5, 59. doi: 10.3390/jof5030059 31288475 PMC6787740

[B8] Cornely O. A. Alastruey-Izquierdo A. Arenz D. Chen S. C. A. Dannaoui E. Hochhegger B. . (2019). Global guideline for the diagnosis and management of mucormycosis: an initiative of the European Confederation of Medical Mycology in cooperation with the Mycoses Study Group Education and Research Consortium. Lancet Infect. Dis. 19, e405–e421. doi: 10.1016/S1473-3099(19)30312-3 31699664 PMC8559573

[B9] Cramer R. A. Rivera A. Hohl T. M. (2011). Immune responses against Aspergillus fumigatus: what have we learned? Curr. Opin. Infect. Dis. 24, 315–322. doi: 10.1097/QCO.0b013e328348b159 21666456 PMC3733365

[B10] Danion F. Rouzaud C. Duréault A. Poirée S. Bougnoux M.-E. Alanio A. . (2019). Why are so many cases of invasive aspergillosis missed? Med. Mycol. 57, S94–S103. doi: 10.1093/mmy/myy081 30816963

[B11] Donnelly J. P. Chen S. C. Kauffman C. A. Steinbach W. J. Baddley J. W. Verweij P. E. . (2020). Revision and update of the consensus definitions of invasive fungal disease from the European Organization for Research and Treatment of Cancer and the Mycoses Study Group Education and Research Consortium. Clin. Infect. Dis. 71, 1367–1376. doi: 10.1093/cid/ciz1008 31802125 PMC7486838

[B12] Gouzien L. Che D. Cassaing S. Lortholary O. Letscher-Bru V. Paccoud O. . (2024). Epidemiology and prognostic factors of mucormycosis in France, (2012-2022): a cross-sectional study nested in a prospective surveillance programme. Lancet Reg. Health Eur. 45, 101010. doi: 10.1016/j.lanepe.2024.101010 39220434 PMC11363841

[B13] Hosseini S. M. S. Borghei P. (2005). Rhinocerebral mucormycosis: pathways of spread. Eur. Arch. Otorhinolaryngol. 262, 932–938. doi: 10.1007/s00405-005-0919-0 15891927

[B14] Jeong W. Keighley C. Wolfe R. Lee W. L. Slavin M. A. Kong D. C. M. . (2019). The epidemiology and clinical manifestations of mucormycosis: a systematic review and meta-analysis of case reports. Clin. Microbiol. Infect. 25, 26–34. doi: 10.1016/j.cmi.2018.07.011 30036666

[B15] Kashyap S. Bernstein J. Ghanchi H. Bowen I. Cortez V. (2019). Diagnosis of rhinocerebral mucormycosis by treatment of cavernous right internal carotid artery occlusion with mechanical thrombectomy: special case presentation and literature review. Front. Neurol. 10, 264. doi: 10.3389/fneur.2019.00264 30972005 PMC6443639

[B16] Kassotis A. Coombs A. Matari N. Lignelli A. Kazim M. (2025). The algorithmic role of critical radiographic features in the treatment of angioinvasive fungal sinusitis. Ophthalmic Plast. Reconstr. Surg. 41, 1–7. doi: 10.1097/IOP.0000000000002783 39240228

[B17] Kontoyiannis D. P. Walsh T. J. (2026). Mucormycosis. N. Engl. J. Med. 394, 684–698. doi: 10.1056/NEJMra2412565 41671483

[B19] Lanternier F. Poiree S. Elie C. Garcia-Hermoso D. Bakouboula P. Sitbon K. . (2015). Prospective pilot study of high-dose (10 mg/kg/day) liposomal amphotericin B (L-AMB) for the initial treatment of mucormycosis. J. Antimicrob. Chemother. 70, 3116–3123. doi: 10.1093/jac/dkv236 26316385

[B18] La Porte C. Provost C. Coste A. Herbrecht R. Denis B. Canet E. . (2026). Multicenter analysis of general presentations and imaging features in cerebral MUCORmycosis (MAGICMUCOR): toward different entities. Med. Mycol. 64, myaf122. doi: 10.1093/mmy/myaf122 41432290

[B20] Latgé J.-P. Chamilos G. (2019). Aspergillus fumigatus and aspergillosis in 2019. Clin. Microbiol. Rev. 33, e00140-18. doi: 10.1128/CMR.00140-18 31722890 PMC6860006

[B21] Leroy J. Vuotto F. Le V. Cornu M. François N. Marceau L. . (2020). Invasive rhino-orbital-cerebral aspergillosis in an immunocompetent patient. J. Mycol. Med. 30, 101002. doi: 10.1016/j.mycmed.2020.101002 32507472

[B22] Lersy F. Royer-Leblond J. Lhermitte B. Chammas A. Schneider F. Hansmann Y. . (2022). Cerebral mucormycosis: neuroimaging findings and histopathological correlation. J. Neurol. 269, 1386–1395. doi: 10.1007/s00415-021-10701-8 34240320

[B23] Li Z. Denning D. W. (2023). The impact of corticosteroids on the outcome of fungal disease: a systematic review and meta-analysis. Curr. Fungal Infect. Rep. 17, 54–70. doi: 10.1007/s12281-023-00456-2 36852004 PMC9947451

[B24] Liang M. Xu J. Luo Y. Qu J. (2024). Epidemiology, pathogenesis, clinical characteristics, and treatment of mucormycosis: a review. Ann. Med. 56, 2396570. doi: 10.1080/07853890.2024.2396570 39221718 PMC11370679

[B25] Little J. S. Cheng M. P. Hsu L. Corrales C. E. Marty F. M. (2019). Invasive fungal carotiditis: a rare manifestation of cranial invasive fungal disease: case series and systematic review of the literature. Open Forum Infect. Dis. 6, ofz392. doi: 10.1093/ofid/ofz392 31660355 PMC6790399

[B26] Meena D. S. Kumar D. Bohra G. K. Kumar G. (2021). Clinical manifestations, diagnosis, and treatment outcome of CNS aspergillosis: a systematic review of 235 cases. Infect. Dis. Now 51, 654–660. doi: 10.1016/j.idnow.2021.04.002 33964485

[B27] Pagano L. Akova M. Dimopoulos G. Herbrecht R. Drgona L. Blijlevens N. (2011). Risk assessment and prognostic factors for mould-related diseases in immunocompromised patients. J. Antimicrob. Chemother. 66 Suppl 1, i5–14. doi: 10.1093/jac/dkq437 21177404

[B28] Pai V. Sansi R. Kharche R. Bandili S. C. Pai B. (2021). Rhino-orbito-cerebral mucormycosis: pictorial review. Insights Imaging 12, 167. doi: 10.1186/s13244-021-01109-z 34767092 PMC8587501

[B29] Rammaert B. Desjardins A. Lortholary O. (2012). New insights into hepatosplenic candidosis, a manifestation of chronic disseminated candidosis. Mycoses 55, e74–e84. doi: 10.1111/j.1439-0507.2012.02182.x 22360318

[B30] Roxbury C. R. Smith D. F. Higgins T. S. Lee S. E. Gallia G. L. Ishii M. . (2017). Complete surgical resection and short-term survival in acute invasive fungal rhinosinusitis. Am. J. Rhinol. Allergy 31, 109–116. doi: 10.2500/ajra.2017.31.4420 28452707

[B31] Safder S. Carpenter J. S. Roberts T. D. Bailey N. (2010). The "Black Turbinate" sign: an early MR imaging finding of nasal mucormycosis. AJNR Am. J. Neuroradiol. 31, 771–774. doi: 10.3174/ajnr.A1808 19942703 PMC7964235

[B32] Serris A. Benzakoun J. Danion F. Porcher R. Sonneville R. Wolff M. . (2022). Cerebral aspergillosis in the era of new antifungals: the CEREALS national cohort study nationwide CEREbral Aspergillosis Lesional study (CEREALS). J. Infect. 84, 227–236. doi: 10.1016/j.jinf.2021.11.014 34838593

[B33] Siddiqui A. A. Shah A. A. Bashir S. H. (2004). Craniocerebral aspergillosis of sinonasal origin in immunocompetent patients: clinical spectrum and outcome in 25 cases. Neurosurgery 55, 602–611. doi: 10.1227/01.neu.0000134597.94269.48 15335427

[B34] Singh G. Vishnu V. Y. (2021). Neurological manifestations of rhino-oculo-cerebral mucormycosis in the COVID-19 era. Nat. Rev. Neurol. 17, 657–658. doi: 10.1038/s41582-021-00560-2 34480144 PMC8414018

[B35] Soare A. Y. Watkins T. N. Bruno V. M. (2020). Understanding mucormycoses in the age of "omics. Front. Genet. 11, 699. doi: 10.3389/fgene.2020.00699 32695145 PMC7339291

[B36] Soliman S. S. M. Baldin C. Gu Y. Singh S. Gebremariam T. Swidergall M. . (2021). Mucoricin is a ricin-like toxin that is critical for the pathogenesis of mucormycosis. Nat. Microbiol. 6, 313–326. doi: 10.1038/s41564-020-00837-0 33462434 PMC7914224

[B333] Surbhi A. Kumar B. Sinha V . (2025) Angioinvasive mucormycosis and internal carotid artery thrombosis: a case series. Indian J. Otolaryngol Head Neck Surg. 77 (1), 536–540. doi: 10.1007/s12070-024-05170-0 PMC11890389 40066397

[B37] Susianti H. Parmadi L. Firani N. K. Setyawan U. A. Sartono T. R. (2021). Diagnostic value of serum human Galactomannan aspergillus antigen and 1,3-beta-D-glucan in immunocompromised patient suspected fungal infection. J. Clin. Lab. Anal. 35, e23806. doi: 10.1002/jcla.23806 33945177 PMC8183930

[B38] Sweed A. H. Anany A. M. Hussein A. Nada W. Eesa M. Elnashar I. . (2025). Endoscopic orbital clearance/debridement: a potential substitute for orbital exenteration in rhino-orbital mucormycosis. Int. Arch. Otorhinolaryngol. 29, 1–7. doi: 10.1055/s-0044-1791645 39850501 PMC11756954

[B39] Tripathi M. Mohindra S. (2018). Rhinocerebral aspergillosis. Lancet 392, e8. doi: 10.1016/S0140-6736(18)31949-4 30215384

[B40] Wei E. Niu J. Zhang M. Zhang Y. Yan K. Fang X. . (2024). Metagenomic next-generation sequencing could play a pivotal role in validating the diagnosis of invasive mold disease of the central nervous system. Front. Cell. Infect. Microbiol. 14, 1393242. doi: 10.3389/fcimb.2024.1393242 38912204 PMC11190073

[B41] Zia Z. Sajadi M. J. Bazrafshan H. Khademi B. Janipour M. (2025). Survival and prognostic factors in rhino-orbito-cerebral mucormycosis: a 3-year cohort study. Sci. Rep. 15, 16088. doi: 10.1038/s41598-025-98926-9 40341683 PMC12062481

