# Supplementary material for: Angioinvasion severity and admission GCS predict outcomes in invasive central nervous system fungal infections
Source: Front Cell Infect Microbiol. 2026 Aug 13;16:1915699. doi: 10.3389/fcimb.2026.1915699 (PMC13518185; doi:10.3389/fcimb.2026.1915699)
Supplement: Supplementary file 1 [file Table2.docx]

Supplementary Material

# 1 Supplementary Table

***Supplementary Table 2. Distribution of positive diagnostic results by specimen type and infection phenotype.***

| **Diagnostic method** | **Specimen type** | **Overall**  N = 57 | **ROCM**  N = 28 | **Rhino-cerebral Aspergillosis**  N = 13 | **Cerebral Aspergillosis**  N = 16 |
| --- | --- | --- | --- | --- | --- |
| Tissue culture | Sinonasal tissue | 1/33 (3%) | 0/21 (0%) | 1/11 (9.1%) | 0/1 (0%) |
|  | Brain tissue | 1/3 (33.3%) | 0/1 (0%) | 0/0 | 1/2 (50%) |
|  | Sinus aspirate | 2/3 (66.7%) | 2/3 (66.7%) | 0/0 | 0/0 |
| Histopathology | Sinonasal tissue | 27/35 (77.1%) | 21/23 (91.3%) | 6/11 (54.5%) | 0/1 (0%) |
|  | Brain tissue | 3/3 (100%) | 1/1 (100%) | 0/0 | 2/2 (100%) |

Supplementary Table 3. Baseline characteristics stratified by 90-day functional outcome

| **Variable** | **N** | **Overall**  N = 57^1^ | **Favorable outcome (mRS 0–2)**  N = 39^1^ | **Unfavorable outcome (mRS 3–6)**  N = 18^1^ | ***p*-value**^2^ |
| --- | --- | --- | --- | --- | --- |
| **Infection group** | 57 |  |  |  | **0.012** |
| *ROCM* |  | 28 (49%) | 14 (36%) | 14 (78%) |  |
| *Rhino-cerebral Aspergillosis* |  | 13 (23%) | 12 (31%) | 1 (5.6%) |  |
| *Cerebral Aspergillosis* |  | 16 (28%) | 13 (33%) | 3 (17%) |  |
| **Age (years)** | 57 | 61.0 (49.0-69.0) | 62.0 (48.0-69.0) | 60.5 (58.0-69.0) | 0.810 |
| **Sex** | 57 |  |  |  | 0.556 |
| *Female* |  | 21 (37%) | 13 (33%) | 8 (44%) |  |
| *Male* |  | 36 (63%) | 26 (67%) | 10 (56%) |  |
| **Diabetes mellitus** | 57 | 41 (72%) | 26 (67%) | 15 (83%) | 0.225 |
| **Diabetic ketoacidosis** | 57 | 10 (18%) | 4 (10%) | 6 (33%) | 0.058 |
| **Definite immunosuppression** | 57 | 7 (12%) | 4 (10%) | 3 (17%) | 0.667 |
| **Consciousness disturbance** | 57 | 19 (33%) | 7 (18%) | 12 (67%) | **<0.001** |
| **Hemiplegia** | 57 | 15 (26%) | 6 (15%) | 9 (50%) | **0.010** |
| **Admission GCS** | 57 | 15.0 (12.0-15.0) | 15.0 (15.0-15.0) | 10.0 (7.0-15.0) | **<0.001** |
| **HbA1c (%)** | 53 | 8.6 (6.2-10.6) | 7.1 (6.1-9.7) | 10.5 (9.0-12.2) | **0.009** |
| **Lymphocyte count (×10⁹/L)** | 57 | 1.2 (0.9-1.7) | 1.2 (1.0-1.7) | 1.0 (0.7-1.3) | **0.021** |
| **CRP (mg/L)** | 55 | 32.8 (8.8-103.7) | 22.3 (4.8-62.3) | 97.9 (20.9-136.1) | **0.020** |
| **Angio score category** | 57 |  |  |  | **<0.001** |
| *None* |  | 30 (53%) | 26 (67%) | 4 (22%) |  |
| *Mild* |  | 9 (16%) | 7 (18%) | 2 (11%) |  |
| *Moderate-severe* |  | 18 (32%) | 6 (15%) | 12 (67%) |  |
| **Orbital involvement** | 57 | 31 (54%) | 18 (46%) | 13 (72%) | 0.089 |
| **Cerebral infarction** | 57 | 23 (40%) | 11 (28%) | 12 (67%) | **0.009** |
| **Internal carotid artery involvement** | 57 | 18 (32%) | 8 (21%) | 10 (56%) | **0.014** |
| **Cavernous sinus involvement** | 57 | 23 (40%) | 12 (31%) | 11 (61%) | **0.043** |
| **Surgery performed** | 57 | 34 (60%) | 27 (69%) | 7 (39%) | **0.043** |
| **Any amphotericin B therapy** | 57 | 27 (47%) | 15 (38%) | 12 (67%) | 0.086 |
| **Onset-to-antifungal interval (days)** | 57 | 21.0 (12.0-45.0) | 29.0 (13.0-62.0) | 15.5 (10.0-21.0) | **0.029** |

^1^n (%); Median (Q1-Q3)

^2^Fisher's exact test; Wilcoxon rank sum test

Abbreviations: GCS, Glasgow Coma Scale; CRP, C-reactive protein.

Supplementary Table 4. Ordinal logistic regression analysis for 90-day functional outcome shift

| **Variable** | **Common OR (95% CI)** | ***p*-value** |
| --- | --- | --- |
| **Infection group** |  |  |
| ROCM | Reference | — |
| Rhino-cerebral Aspergillosis | 0.72 (0.20–2.55) | 0.610 |
| Cerebral Aspergillosis | 0.33 (0.08–1.30) | 0.112 |
| **Admission GCS** | 0.73 (0.56–0.95) | 0.020 |
| **Angioinvasion severity** |  |  |
| None | Reference | — |
| Mild angioinvasion | 2.36 (0.55–10.15) | 0.247 |
| Moderate-severe angioinvasion | 8.61 (1.87–39.58) | 0.006 |
| **Lymphocyte count** | 0.61 (0.36–1.03) | 0.062 |

Abbreviations: OR, odds ratio; CI, confidence interval; GCS, Glasgow Coma Scale; ROCM, rhino-orbital-cerebral mucormycosis.

# 2 Supplementary Figure

**Supplementary Figure 1. Restricted cubic spline analysis of continuous predictors for mortality during follow‑up.**

(A) Association between admission GCS score and adjusted hazard ratio for mortality.

(B) Association between baseline lymphocyte count and adjusted hazard ratio for mortality.

The solid line represents adjusted hazard ratios derived from the multivariable Cox proportional hazards model, and the shaded area indicates the 95% confidence interval.

**Supplementary Figure 2. Multivariable regression analyses for 180-day neurological functional outcomes.**

(A) Forest plot showing adjusted ORs and 95% CIs for predictors of unfavorable functional outcome (mRS score ≥3) at 180 days derived from the multivariable Firth penalized logistic regression model, which was used to reduce small-sample bias and address potential separation issues. OR >1 indicates higher odds of unfavorable functional outcome.

(B) Forest plot showing common ORs and 95% CIs derived from the proportional odds ordinal logistic regression model using the full 180-day mRS distribution (0–6). Common OR >1 indicates a shift toward worse neurological disability across the entire mRS spectrum.
